# Smad3 gene C‐terminal phosphorylation site mutation aggravates CCl_4_‐induced inflammation in mice

**DOI:** 10.1111/jcmm.15385

**Published:** 2020-05-14

**Authors:** Hanyan Ding, Meng Fang, Yongfang Gong, Dong Li, Chong Zhang, Guanghua Wen, Chao Wu, Jingjing Yang, Yan Yang

**Affiliations:** ^1^ Department of Pharmacology Key Laboratory of Anti‐inflammatory and Immunopharmacology Ministry of Education Anhui Medical University Hefei China; ^2^ Department of Anatomy Anhui Medical University Hefei China

**Keywords:** inflammation, liver, mutation, pSmad3C

## Abstract

The expression of C‐terminal phosphorylated Smad3 (pSmad3C) is down‐regulated with the progression of liver disease. Thus, we hypothesized that pSmad3C expression may be negatively related to liver disease. To develop novel therapeutic strategies, a suitable animal model is required that will allow researchers to study the effect of Smad3 domain‐specific phosphorylation on liver disease progression. The current study aimed to construct a new mouse model with the Smad3 C‐terminal phosphorylation site mutation and to explore the effects of this mutation on CCl_4_‐induced inflammation. Smad3 C‐terminal phosphorylation site mutant mice were generated using TetraOne™ gene fixed‐point knock‐in technology and embryonic stem cell microinjection. Resulting mice were identified by genotyping, and the effects on inflammation were explored in the presence or absence of CCl_4_. No homozygous mice were born, indicating that the mutation is embryonic lethal. There was no significant difference in liver phenotype and growth between the wild‐type (WT) and heterozygous (HT) mice in the absence of reagent stimulation. After CCl_4_‐induced acute and chronic liver damage, liver pathology, serum transaminase (ALT/AST) expression and levels of inflammatory factors (IL‐6/TNF‐α) were more severely altered in HT mice than in WT mice. Furthermore, pSmad3C protein levels were lower in liver tissue from HT mice. These results suggest that Smad3 C‐terminal phosphorylation may have a protective effect during the early stages of liver injury. In summary, we have generated a new animal model that will be a novel tool for future research on the effects of Smad3 domain‐specific phosphorylation on liver disease progression.

## 1 INTRODUCTION

TGF‐β is an intracellular signalling regulator involved in the development and progression of several diseases, including acute/chronic liver disease, liver fibrosis, and cancer; it is primarily transmitted by Smad transcription factors.^1^, ^2^ Smad3 is the major signalling molecule for the TGF‐β/Smad pathway in cells.^3^, ^4^, ^5^ With the development of specific antibodies, activated forms of the Smad3 protein are being discovered, which include C‐terminal phosphorylated Smad3 (pSmad3C), linker region phosphorylated Smad3 (pSmad3L) and double phosphorylated Smad3 (pSmad3L/C).^6^, ^7^, ^8^ When TGF‐β_1_ binds to the membrane receptor, activated type I receptors (TβRI) phosphorylate the C‐terminus of Smad2 or Smad3 to form pSmad2C and pSmad3C.^9^ These then form hetero‐oligomers with Smad4, a common partner, and then translocate to the nucleus where they regulate target gene transcription^10^, ^11^, ^12^ and produce growth inhibition signals.^13^, ^14^ TGF‐β also activates the mitogen‐activated protein kinase (MAPK) pathway, which alters phosphorylated Smad3 signalling, increases mitogenic pSmad3L activity and decreases TGF‐β‐dependent cytostatic pSmad3C.^15^, ^16^, ^17^ Clinical evidence has shown that during the development of liver cancer from chronic hepatitis B, pSmad3L levels gradually increase, while pSmad3C decreases.^7^, ^18^, ^19^ After successful anti‐hepatitis virus treatment, pSmad3C levels in hepatocytes returned to normal, while pSmad3L was reduced.^18^ In addition, during the development of liver fibrosis‐hepatocarcinoma, compound *Astragalus* and *Salvia miltiorrhiza* extracts were shown to promote pSmad3C and inhibit pSmad3L to suppress hepatocarcinogenesis.^20^ Therefore, the TGF‐β_1_/Smad3 pathway can not only inhibit hepatocyte growth but also promote the development of liver fibrosis and cancer, meaning that it inhibits tumour cell proliferation and also promotes mitosis. Interestingly, this dual effect is known to be associated with different Smad3 phosphorylation sites^8^, ^21^, ^22^, ^23^; however, there have been few reports on the role of domain‐specific Smad3 phosphorylation in the development of liver disease, and the underlying mechanism also remains to be explored.

Animal models are indispensable for studying the pathogenesis of acute and chronic liver disease, and for understanding the mechanism of action of specific genes during the development of liver disease. These include animal models induced by hepatotoxic agents, transplanting tumour cells into animals and genetic engineering.^24^ Genetically engineered animals provide an ideal experimental model for medical experimental research. In addition to allowing research into disease progression at the animal and tissue level, they can also deepen our understanding of disease pathogenesis at the cellular and molecular level for drug screening and pre‐clinical studies. Knock‐in technology has been used to delete endogenous genomic regions and to induce spontaneous mutations by targeted nucleotide substitution.^25^ Embryonic stem (ES) cell gene targeting technology is an experimental means to alter the genetic information of living organisms via homologous recombination. The coding gene fragment is microinjected into ES cells in vitro and is integrated via homologous recombination so that it becomes heritable. Animals that are homozygous for the mutated gene can then be generated by breeding. The process of homologous recombination combined with ES cell microinjection technology makes it possible to introduce coding genes into mice and can generate mutant animals at a speed unmatched by conventional experimental methods.^24^

Smad3‐deficient mice are prone to cancer, including colon cancer and skin cancer. This deficiency can also cause immune disorders, infection, osteoarthritis and ultimately premature death 1‐10 months after birth.^26^ Furthermore, Smad3 gene deficiency can affect immune regulation, promote inflammation and drive cancer progression. Smad3 plays a complex role in the transduction of various signals in the body.^27^, ^28^ Unfortunately, a complete loss of Smad3 causes many side effects, and we therefore could not use this as an model animal to study the molecular mechanisms of liver disease progression. We therefore hypothesized that mice in which only pSmad3C is mutated may be more susceptible to liver disease. Thus, we selectively up‐regulated pSmad3C/3L in HepG2 cells via plasmid transfection. Interestingly, we found that overexpression of pSmad3C promoted apoptosis and inhibited cell proliferation and migration, whereas overexpression of the pSmad3L protein promoted cell proliferation and migration and inhibited apoptosis.^29^ These results suggest that domain‐specific phosphorylation of Smad3 at the cellular level is closely associated with the occurrence of liver cancer. The next step would then be to determine the in vivo effects of a pSmad3C mutation. Thus, to further investigate the role of Smad3 C‐terminal phosphorylation in the development of liver disease, we generated a mouse model that expresses low levels of pSmad3C. The *Smad3* gene is located on mouse chromosome 9 and comprises nine exons. The ATG start codon is located in exon 1, and the TAG stop codon is in exon 9. Smad3 C‐terminal serine residues Ser422/423/425 are located in exon 9, and the residues at position 423 and 425 can be phosphorylated.^6^, ^10^, ^30^ The aim of the current study was to mutate these three sites using TetraOne™ gene fixed‐point knock‐in technology. The classical genetic engineering technology‐ES cell microinjection technique and the principle of homologous recombination were used to generate mice carrying a mutation in the C‐terminal phosphorylation site of the *Smad3* gene. We then explored the effects of these separately in the presence or absence of CCl_4_ stimulation.

## 2 MATERIALS AND METHODS

### 2.1 Generation of Smad3 C‐terminal phosphorylation site mutant mice

C57BL/6 ES cells were used for gene targeting. Wild‐type C57BL/6 mice were provided by Cyagen Biosciences Co., Ltd, License No. SCXY (Guangdong) 2013‐0032, certificate No. 44410400001282, specific‐pathogen free (SPF) environment. *S422A* (TCC to GCC), *S423A* (AGT to GCT) and *S425A* (TCT to GCT) mutations were introduced into exon 9 using 3′ homology arms. To engineer the targeting vector, homology arms were generated by PCR using BAC clones RP24‐398N4 and RP23‐349G12 from the C57BL/6 library as a template. In the targeting vector, a neo cassette was flanked by loxP sites. DTA was used for negative selection. DNA fragments were constructed with three site mutations at positions 422, 424 and 425 of the *Smad3* gene C‐terminal, such that homologous recombination would lead to the insertion and replacement of the gene of interest at the specific position on the chromosome. Southern blotting was used to screen for positive ES cells, which were then injected into blastocysts derived from surrogate mother mice. F0 mice (containing the neo cassette) were obtained and then mated with wild‐type (WT) C57BL/6 mice to generate F1 progeny. Genotypes were determined by polymerase chain reaction (PCR) and gene sequencing of tail DNA. This generated 10 heterozygous (HT) mice: 6 males and 4 females.

### 2.2 Animals

All procedures were approved by the Animal Experimental Ethics Committee of Anhui Medical University. Heterozygous (HT) and wild‐type (WT) mice were raised and propagated under SPF conditions at the Center for Laboratory Animal Science at Anhui Medical University [Certificate No. SYXK(Anhui)2017‐006]. Mice were kept on standard food and allowed to access food and water ad libitum. Genotypes of new progeny were determined at 3 weeks of age by PCR analysis of tail DNA. Male and female mice reached breeding age at 60‐90 days, and breeding was carried out at a male:female ratio of 1:2 cohabitation. Female mice became pregnant about 21 days later and were given appropriate amounts of autoclaved seeds and eggs to provide nutrition during pregnancy.

### 2.3 Genotyping

DNA was extracted from a 0.3‐0.5 cm piece of tail. Samples were incubated in 150 μL solution A (160 mg NaOH, 12 mg EDTA, 200 mL ddH_2_O) for 45 minutes at 95°C in a water bath. After denaturation, samples were cooled to room temperature before adding 150 μL solution B (10 mL 1.0 mol/L Tris‐HCl, 240 mL ddH_2_O) and mixing. Samples were centrifuged at 13684.32 *g* for 2 minutes, and the supernatant containing the DNA was transferred to a sterile tube. Samples were stored at −20°C until PCR. A PCR mix containing 12.5 μL 2× Taq PCR master mix, 9 μL ddH_2_O and 1 μL of each primer (1 mol/L) [Forward: 5′‐TATGTCGCCACAGCAGATAGCC‐3′; reverse: 5′‐CAGCTGTACTGACATGCCTGTCTG‐3′ (Sangon Biotech)] was added to each well of a PCR plate (total volume 23.5 μL), followed by 1.5 μL DNA. The amplification reaction was carried out using a T100™ Thermal cycler system (Bio‐Rad). DNA was initially denatured for 3 minutes at 94°C, then amplified for 32 cycles: denaturation at 94°C for 30 seconds, annealing at 62°C for 35 seconds and extension at 72°C for 35 seconds, followed by a final extension for 5 minutes at 72°C. Amplified samples were stored at 4°C or at −20°C for long‐term storage. Agarose gel electrophoresis was performed to determine the genotypes. Amplified samples were also sent to Sangon Biotech Co., Ltd. for gene sequencing.

### 2.4 Preliminary study on the phenotype of heterozygous mice under standard conditions and following acute liver injury

The number of foetuses and progeny of each genotype was recorded. Bodyweight, growth status and activity were also recorded from birth. At 6 weeks of age, WT and HT male mice were treated with 0.1% CCl_4_ (10 mL/kg; diluted with corn oil) twice a week via intraperitoneal injection. Control mice were given corn oil alone (10 mL/kg). Mice were killed 24 hours or 2 weeks later. Serum samples were collected before killing and used for transaminase and inflammatory‐factor kit assays. After killing, the liver was isolated, and a portion was fixed in 4% paraformaldehyde for 24 hours. Samples were embedded in paraffin, and sections were cut at 5 µm, and either stained with haematoxylin and eosin (HE) or used for immunofluorescence. The remaining liver tissue was snap‐frozen in liquid nitrogen and stored at −80°C for Western blot analysis.

### 2.5 Haematoxylin and eosin (H&E) staining

Liver tissue was embedded in paraffin and cut into 5‐μm sections for H&E. Briefly, tissue sections were stained in haematoxylin, washed in running water to develop the blue colour and then stained in eosin before mounting under coverslips. Pathological features were assessed. Photoshop 7.0 image processing software performs semi‐quantitative analysis of necrotic and degenerative tissue areas on pathological pictures. Liver damage proportion (%) = liver tissue damage area (pixels)/photographs area (pixels) × 100%.

### 2.6 Immunofluorescence

Antigen retrieval was performed on tissue sections before blocking. Sections were washed with PBS, d then incubated with rhodamine (TRITC)‐conjugated goat anti‐rabbit IgG (ZF‐0316, ZSGB‐BIO) at 37°C for 50 minutes. After washing with PBS, sections were incubated in a DAPI solution (C1005; Beyotime) for 10 minutes. Finally, sections were washed with PBS and mounted with anti‐fade mounting medium (P0126; Beyotime) before being viewed and photographed under a fluorescence microscope (Leica). Images were evaluated using ImageJ software (NIH).

### 2.7 Western blot

Liver tissue was homogenized in cell lysis buffer (P0013; Beyotime). Primary antibodies used were rabbit anti‐p‐Smad3 (Ser423/425) monoclonal (Santa Cruz Biotechnology), rabbit anti‐Smad3 polyclonal (Santa Cruz Biotechnology) and mouse anti‐glyceraldehyde 3‐phosphate dehydrogenase (GAPDH) monoclonal antibodies (Cell Signaling Technology). GAPDH served as an internal control. Results were analysed using ImageJ software.

### 2.8 Statistical analysis

Data are shown as mean ± standard error (SE). Statistical analysis was performed using one‐way analysis of variance or Student's *t* test. Significant differences were defined when *P* < .05.

## 3 RESULTS

### 3.1 Generation of mice carrying a Smad3 C‐terminal phosphorylation site mutation

**Figure 1:**
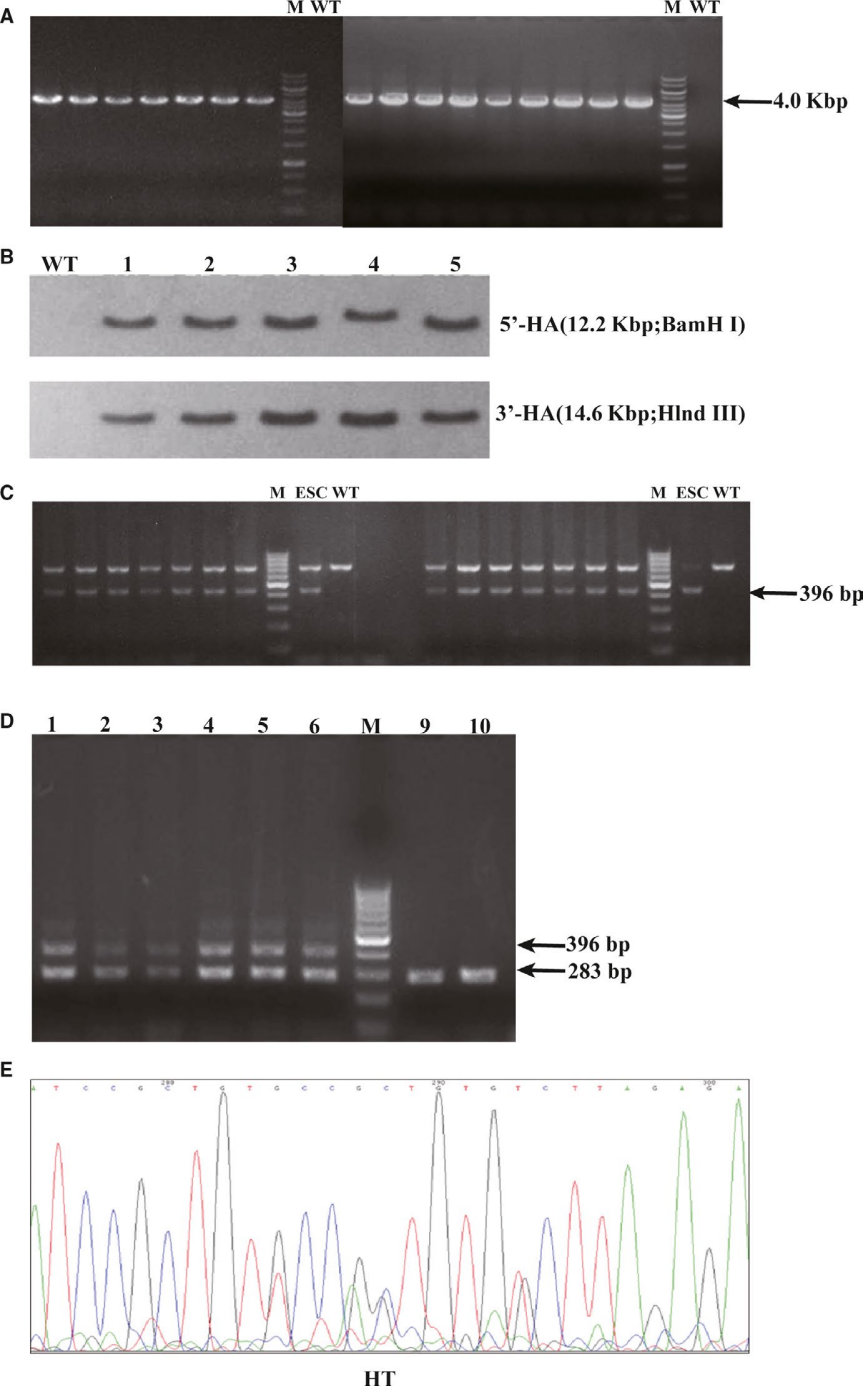
Generation of mice with a Smad3 C‐terminal phosphorylation site mutation. A, Positive ES cell clones were verified by PCR (wild‐type (WT): NA; mutation (MT): 4.0 kbp). B, Southern blot was used to validate five positive clones. C, Sixteen positive F0 chimeric mice were identified by PCR genotyping (MT: 396 bp). D, Agarose gel electrophoresis of PCR products from F1 generation mice (WT: 283 bp MT: 396 bp). E, Peak of PCR product sequencing of F1 generation mice. F1 generation mice were heterozygotes (HT)

According to the design scheme, three serine phosphorylation sites (*Ser422/423/425*) at the C‐terminus of the murine *Smad3* gene were selected as target sites and were recognized by the TetraOne™ gene site‐specific knock‐in technique. C57BL/6 ES cells were used for gene targeting. After transfection of the targeting vector to ES cells, PCR identified 16 positive clones (Figure 1A), 5 of which were selected for validation by Southern blot (Figure 1B). Two positive clones were successfully microinjected into blastocysts and subsequently returned to surrogate mice. The resulting chimeric mice were verified by PCR to obtain positive F0 founders (Figure 1C). These F0 mice were crossed to WT mice to obtain an F1 generation. With this method, we successfully generated 6 mice that carried the Smad3 C‐terminal phosphorylation site mutation (heterozygous, F1 generation). These animals (4 females and 2 males) were transferred to the Center for Laboratory Animal Sciences at Anhui Medical University. Tail DNA was extracted using the acid‐base method, and gene fragments were obtained by PCR. Amplified products were then subjected to agarose gel electrophoresis to identify genotypes (Figure 1D). All genotypes were verified by sequencing (Figure 1E). Results show that all 6 mice were HT for the mutation.

### 3.2 No HO progeny were obtained after breeding mice carrying the Smad3 C‐terminal phosphorylation site mutation

One hundred and fifty progeny were genotyped, and results showed that these comprised 59 WT and 91 HT mice, which is close to the expected 1:2 ratio. No HO progeny were identified. Ten each of WT and HT pregnant females were randomly selected, and the number of newborn mice obtained from each was counted. WT mice gave birth to 7‐9 newborns per litter, and HT mice produced 4‐6 newborns per litter (Table 1).

**Table 1:** Pregnant mice born mice per litter

	WT	HT
Quantity	5.7 ± 0.82	4.9 ± 0.74*

Note

$\bar{x}$ ± SE; n = 10 mice/group.

Abbreviations: HT, heterozygous mice; WT, wild‐type mice.

* *P* < .05 vs WT group.

### 3.3 WT, HT and HO embryos were identified in pregnant HT mice

**Figure 2:**
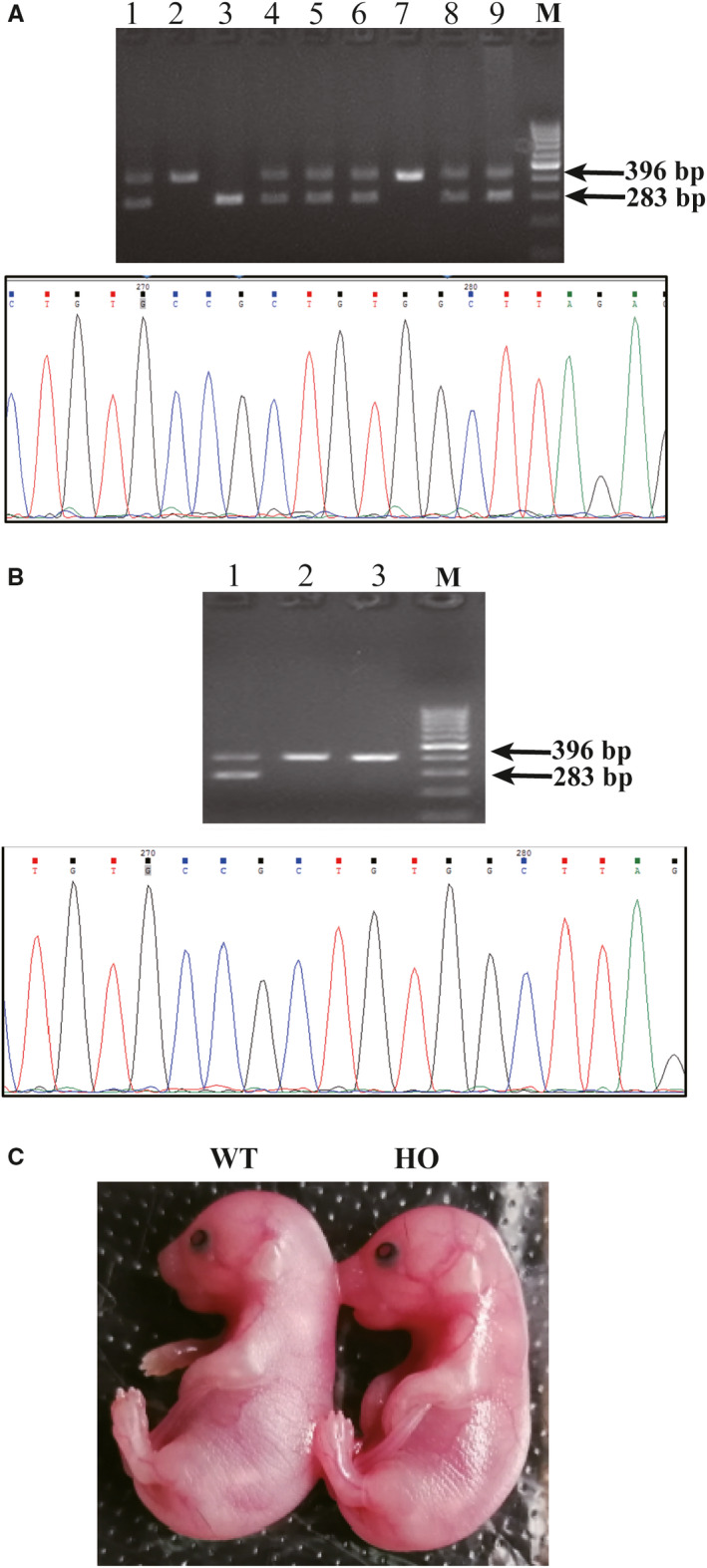
Embryo genotype and phenotype results. A, Genotypes of E14.5 embryos were determined by PCR and gene sequencing (WT: 283 bp; MT: 396 bp). B, Genotypes of E17.5 embryos were determined by PCR and gene sequencing (WT: 283 bp; MT: 396 bp). C, Appearance of homozygous (HO) and WT embryos at E19.5 (left: WT; right: HO)

No HO newborn mice were obtained after breeding the heterozygous mutant mice for more than 1 year. Therefore, we collected embryos from pregnant mice at E14.5, E17.5 and E19.5 to determine their genotype. HO embryos were identified at all stages (Figure 2A,B) and were alive, although slightly smaller than their WT littermates (Figure 2C).

### 3.4 No statistical difference in HT vs WT phenotypes in the absence of reagent stimulation

**Figure 3:**
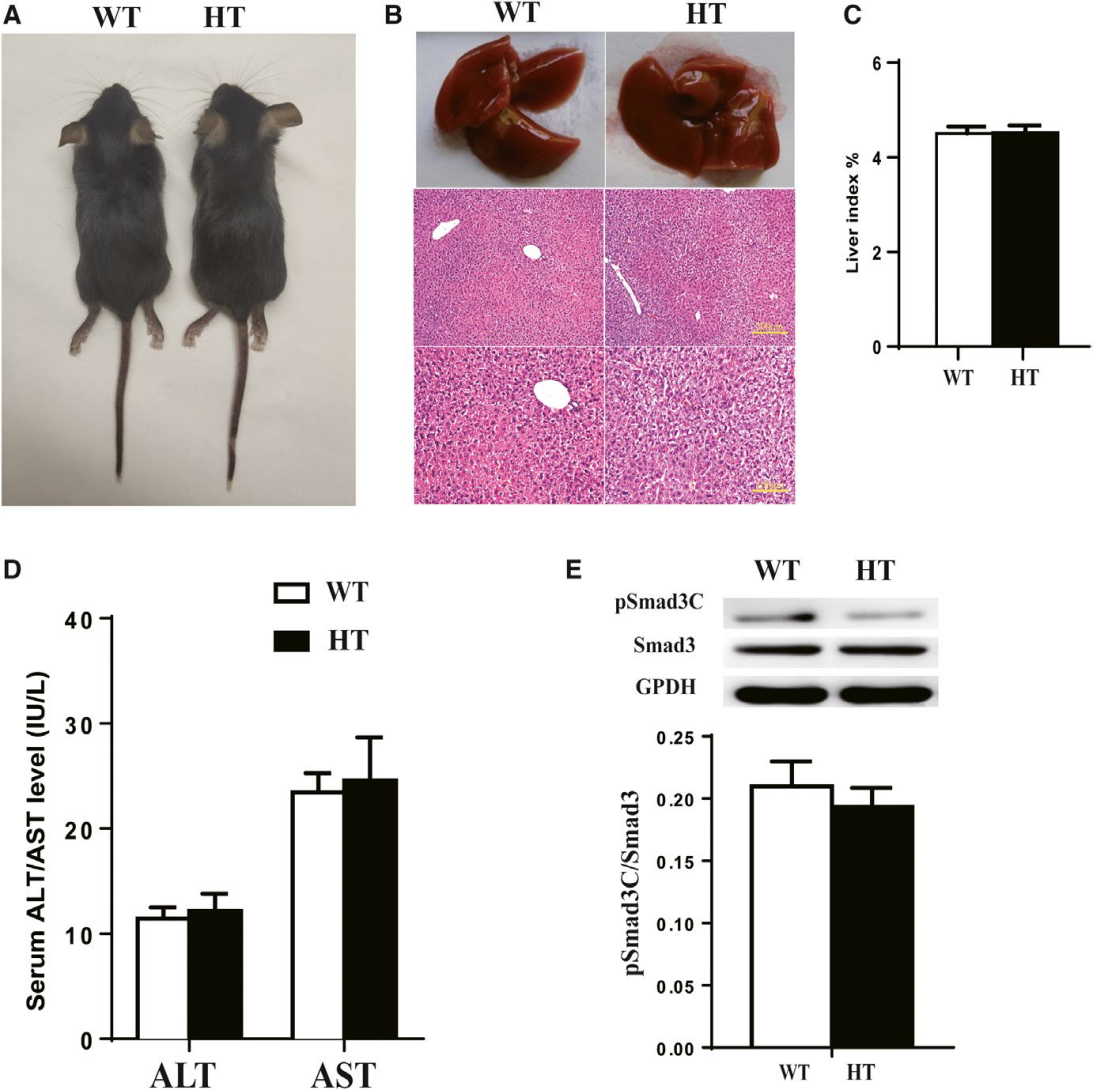
Phenotype of HT mice without reagent stimulation. A, Appearance of HT mice and WT mice (left: WT; right: HT). B, H&E staining for histological analysis of liver tissue. C, Liver index results for HT and WT mice. D, Serum ALT/AST levels in HT and WT mice ( $\bar{x}$ ± SE, n = 6). E, pSmad3C expression levels analysed by Western blot ( $\bar{x}$ ± SE, n = 3). *P* < .01, WT‐CCl_4_ vs WT‐Control; *P* < .01, HT‐CCl_4_ vs HT‐Control; *P* < .05, *P* < .01, HT‐Control vs WT‐CCl_4_

Based on the characteristics of mouse growth and development, we selected several key stages (newborn, 1 week old, 3 weeks old, 5 weeks old and 7 weeks old) to observe and record the appearance and behaviour of HT and WT mice. Newborn mice were ruddy, hairless, and their eyes were closed; one‐week‐old mice had black fur, their eyes were open, and they were uniform in size; three‐week‐old mice displayed basic foraging ability, were able to drink water spontaneously and were active; at five weeks old, mice were near maturity; seven‐week‐old mice were mature and active (Figure 3A). Ten randomly selected C57BL/6 WT mice and HT mice were weighed at three weeks, five weeks and seven weeks of age (Table 2). Results indicate that the appearance and growth of HT mice at different ages was not significantly different compared to WT mice.

**Table 2:** Comparison of mouse bodyweight

Age/wk	WT		HT	
	Female	Male	Female	Male
3 wk	9.1 ± 1.7	10.2 ± 1.5	9.3 ± 1.6	9.2 ± 1.8
5 wk	15.4 ± 2.7	19.6 ± 2.1	15.1 ± 2.3	19.5 ± 2.6
7 wk	17.2 ± 3.2	23.1 ± 3.3	17.4 ± 2.9	22.6 ± 3.1

Note

$\bar{x}$ ± SE; n = 10 mice/group.

Abbreviations: HT, heterozygous mouse; WT, wild‐type mouse.

Male WT and HT mice (n = 6; 6 weeks old) were randomly selected, and serum and liver samples were harvested. The mice were weighed before killing, and the liver was removed and washed with physiological saline. After carefully aspirating water from the surface of the liver, the organ was weighed and photographed. The edge of the liver was clear, the colour was bright red and shiny, and there were no spots or nodules on the surface. There was no significant difference in size between livers from WT and HT mice (Figure 3B). Hepatic pathological sections were stained with H&E (Figure 3B) and showed that cells were neatly arranged and uniform in size, with no lesions of flaky inflammatory cells or obvious areas of necrosis or nuclear fragmentation. The pathological state of HT and WT mice was not obvious. Mouse liver index results showed that there was no significant difference in liver weight between WT and HT mice (Figure 3C). There was no statistical difference in transaminase levels (Figure 3D). Total protein was extracted from fresh liver tissue. The target protein was isolated and incubated with pSmad3C‐ and Smad3‐specific antibodies. Results show that the expression of pSmad3C was lower in HT mice than in WT mice (Figure 3E).

### 3.5 Smad3 C‐terminal phosphorylation site mutation aggravates CCl_4_‐induced acute liver injury

**Figure 4:**
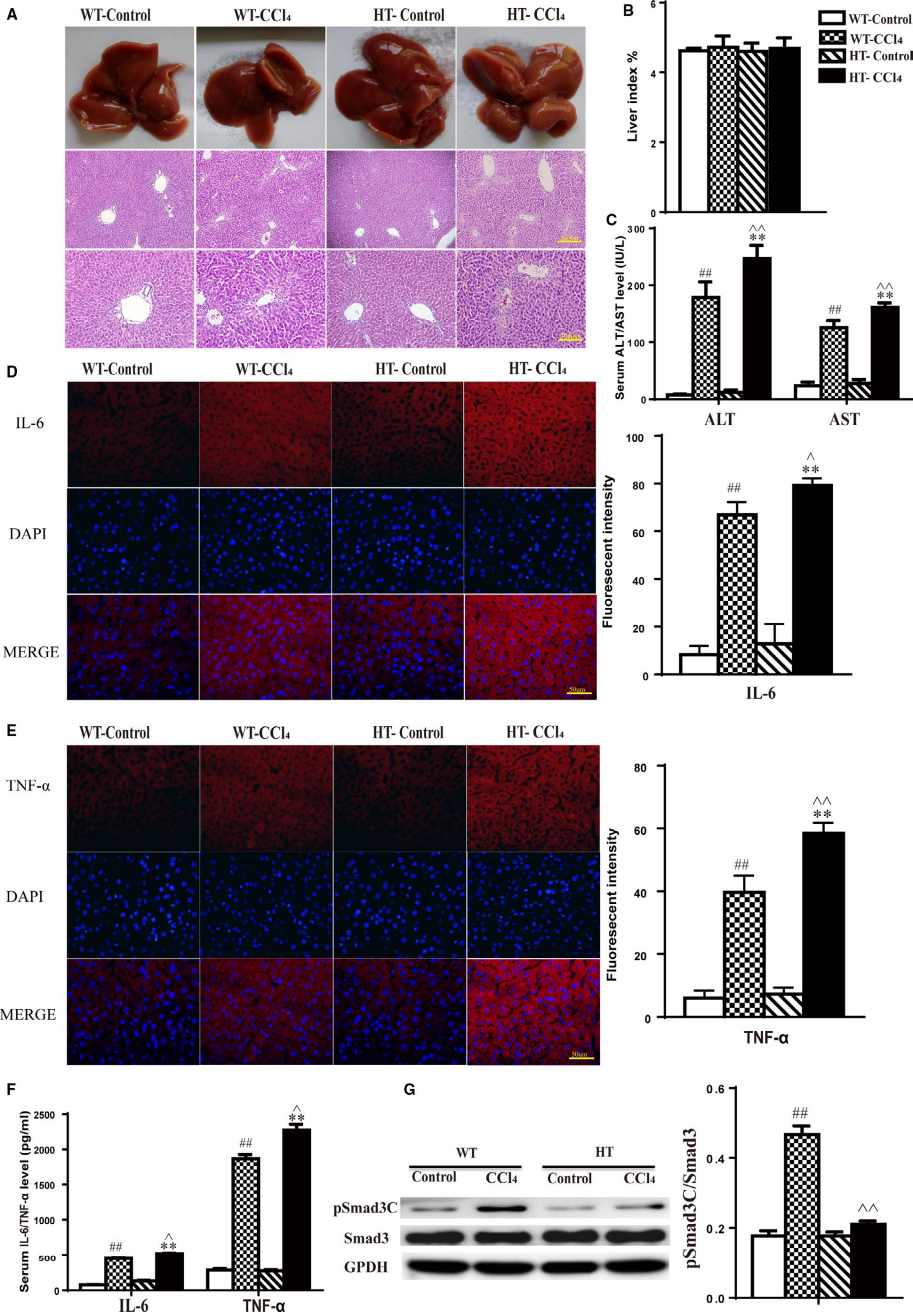
CCl_4_‐induced acute liver injury. A, H&E staining for histological analysis of liver tissue. Magnification, 100× and 200×. B, Liver index results for HT and WT mice. C, Serum ALT/AST levels in HT and WT mice. D, Fluorescence intensity of IL‐6 in the liver, photographed using a fluorescence microscope. E, Fluorescence intensity of TNF‐ɑ in the liver, photographed using a fluorescence microscope. F, Serum IL‐6/TNF‐ɑ levels in HT and WT mice. G, pSmad3C expression levels analysed by Western blot ( $\bar{x}$ ± SE, n = 3). $\bar{x}$ ± SE, n = 6, ^##^ *P* < .01, WT‐CCl_4_ vs WT‐Control; **P < .01, HT‐CCl_4_ vs HT‐Control; ^^^ *P* < .05, ^^^^ *P* < .01, HT‐Control vs WT‐CCl_4_

Twelve HT and WT mice, each, were divided into four groups: WT‐control, WT‐CCl_4_, HT‐control and HT‐CCl_4_. Mice were killed 24 hours after treatment. Within the control group, there was no significant difference in the liver or pathological changes between WT and HT mice (Figure 4A). The livers of HT mice from the CCl_4_ group were rough in appearance, blunt at the edges, greyish‐red in colour, and the surface was covered with grey‐white spots, which were more dense than those in WT mice (Figure 4A). Light microscopy showed that the hepatocytes of HT mice were disordered, the hepatic sinus was larger in size, and the hepatocyte size was also changed. There were visible spots, fragmentary necrosis, inflammatory cell infiltration and balloon‐like changes in the livers of HT mice (Figure 4A). Analysis of the proportion of the liver damage to the total area of the liver HE section showed that the HT group injury area was significantly larger than the WT group (Table 3). In summary, livers from HT mice displayed more severe pathological changes than WT mice. Mouse liver index results showed that there was no significant difference in liver weight between WT and HT mice (Figure 4B).

**Table 3:** Effect of Smad3 C‐terminal phosphorylation site mutation on liver damage proportion in mice induced by CCl_4_ for 24 h

Group	Damage proportion/%
WT‐control	0.0 ± 0.0
WT‐CCl_4_	61.1 ± 6.4^##^
HT‐control	0.0 ± 0.0
HT‐CCl_4_	69.1 ± 3.2**

Note

$\bar{x}$ ± SE; n = 6.

Abbreviations: HT, heterozygous mouse; WT, wild‐type mouse.

^##^ *P* < .01, WT‐CCl_4_ vs WT‐Control.

** *P* < .01, HT‐CCl_4_ vs WT‐ CCl_4_.

Transaminase assays revealed that HT and WT mice in the control group showed no significant differences in serum ALT and AST levels. ALT and AST levels were significantly higher in the CCl_4_ group than in the control group, and within the CCl_4_ group, the levels were higher in HT mice than in WT mice (Figure 4C). In the control group, serum levels of the inflammatory factors IL‐6 and TNF‐α showed no significant differences between HT and WT mice. However, the levels of these two inflammatory factors were significantly higher in the CCl_4_ group than in the control group, and within the CCl_4_ group, the levels were higher in HT mice than in WT mice (Figure 4D‐F).

Total protein was extracted from fresh liver tissue. Target protein was isolated and incubated with pSmad3C‐ and Smad3‐specific antibodies. Results show that there was no significant difference in the Smad3 level in each group, or in the pSmad3C level in the control group. The pSmad3C expression in the livers of WT mice was significantly increased after CCl_4_‐induced acute liver injury, while the pSmad3C expression in HT mice was low (Figure 4G).

### 3.6 Smad3 C‐terminal phosphorylation site mutation aggravates CCl_4_‐induced chronic liver injury

**Figure 5:**
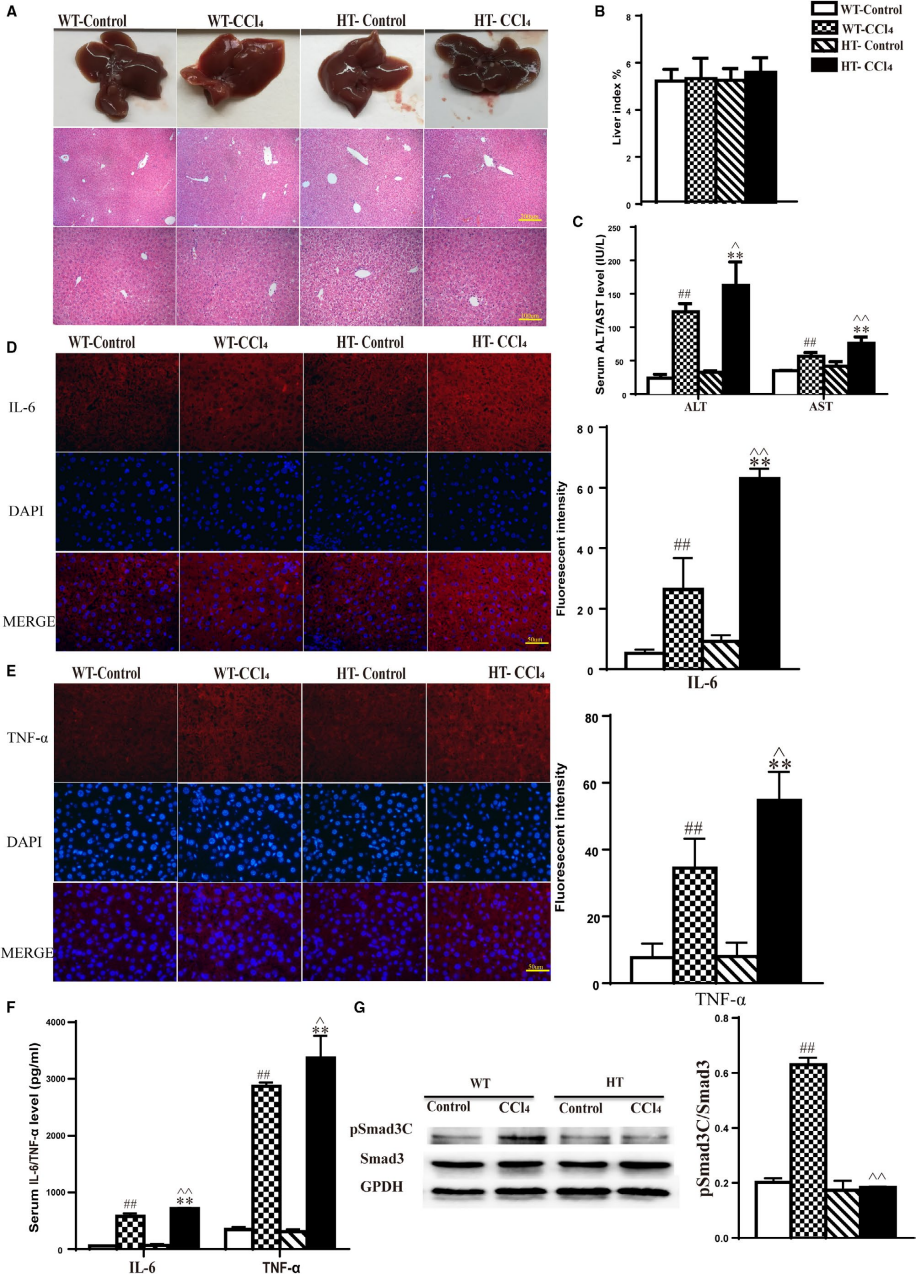
CCl_4_‐induced chronic liver injury. A, H&E staining for histological analysis of liver tissue. Magnification, 100× and 200×. B, Liver index results for HT and WT mice. C, Serum ALT/AST levels in HT and WT mice. D, Fluorescence intensity of IL‐6 in the liver, photographed using a fluorescence microscope. E, Fluorescence intensity of TNF‐ɑ in the liver, photographed using a fluorescence microscope. F, Serum IL‐6/TNF‐ɑ levels in HT and WT mice. G, pSmad3C expression levels analysed by Western blot ( $\bar{x}$ ± SE, n = 3). $\bar{x}$ ± SE, n = 6, ^##^ *P* < .01, WT‐CCl_4_ vs WT‐Control; **P < .01, HT‐CCl_4_ vs HT‐Control; ^^^ *P* < .05, ^^^^ *P* < .01, HT‐Control vs WT‐CCl_4_

Mice were killed 2 weeks after model induction. In the control group, there were no significant differences in pathological changes in the livers of WT and HT mice (Figure 5A). Strikingly, both models showed liver injury, an increase in inflammatory cells and vacuolar‐like hepatocyte lesions; of the two, HT mice were observed to be more sensitive (Figure 5A). Mouse liver index results showed that there was no significant difference in liver weight between WT and HT mice (Figure 5B). ALT and AST levels were significantly increased in HT mice compared to WT mice (Figure 5C). Within the control group, the levels of these two inflammatory factors in the serum and hepatic tissue showed no significant differences between HT and WT mice. In the CCl_4_ group, ALT and AST levels were significantly higher than in the control group, and within the CCl_4_ group, the levels were higher in HT mice than in WT mice (Figure 5D‐F).

Total protein was extracted from fresh liver tissue. The target protein was isolated and incubated with pSmad3C‐ and Smad3‐specific antibodies. Results show that there was no significant difference in the expression of Smad3 in each group or in the expression of pSmad3C in the control group. pSmad3C expression was significantly increased in the livers of WT mice after CCl_4_‐induced chronic liver injury, while the pSmad3C level in HT mice remained low (Figure 5G).

## 4 DISCUSSION

Domain‐specific phosphorylation of the Smad3 protein is known to be closely related to the development of liver disease. In this study, we developed a new animal model in which the Smad3 C‐terminal phosphorylation site was mutated.

Our research team and Cyagen Biosciences Co., Ltd. independently carried out heterozygous crossbreeding, and after almost one year, only HT and WT newborn mice were generated, indicating that the mutation is homozygous lethal. We were, however, able to obtain HO embryos at E14.5, E17.5 and E19.5. At all three stages, the HO embryos were alive but were slightly smaller than the WT littermates. Of the 150 live progeny, the proportion of HT to WT mice was close to 2:1. This is consistent with Mendel's law of inheritance, which states that the expected proportion of progeny from a heterozygous cross is WT:HT:HO = 1:2:1. An assessment of the phenotype of HT mice of different ages showed that there was no significant difference in the appearance or growth of HT mice and WT mice. However, HT mice were significantly reduced in number compared to the WT mice per litter. Previous research showed that mice carrying a constitutive deletion of Smad2 failed to form an organized egg cylinder and lacked the mesoderm, resulting in homozygous embryos dying during the early stages of development.^31^ In addition, Smad4 is considered to be an essential factor in TGF‐β signalling.^32^ Targeted deletion of *Smad4* resulted in abnormal development during the egg cylinder stages and a failure to form the mesoderm, such that homozygotes died during early embryonic development.^33^ Loss of *Smad3* did produce viable homozygous mutant mice; however, as a result of defects in immune function, they died between 1 and 8 months of age.^26^, ^27^, ^28^ *Smad3* is ubiquitously expressed during embryonic development, as well as in various adult organs, suggesting that it may play an important role in vertebrate embryonic growth and organ development. Furthermore, mutations at the C‐terminal phosphorylation site of Smad3 prevent the expression of pSmad3C, and during ontogeny, a single gene can participate in a number of different processes. In summary, it appears that a mutation in the C‐terminal phosphorylation site of Smad3 is homozygous lethal; however, the specific cause requires further investigation.

We also investigated the effects of low pSmad3C expression on the liver and liver disease progression in HT mice. To validate our new animal model, we assessed the phenotype in the presence or absence of CCl_4_ stimulation, and compared the phenotypes of HT and WT mice. Results showed that HT mice with different body index and pSmad3C expression levels were not significantly different compared to WT C57BL/6 mice. After CCl_4_‐induced acute/chronic liver injury, the liver appearance and associated pathological changes were more severe in HT mice, and the increase in serum transaminase (ALT/AST) levels was more obvious than that in WT mice. Serum ALT and AST are often used to assess liver function in clinical and pre‐clinical studies and are typically elevated as a result of liver injury in mice.^34^ In the early stages of liver injury, the increase in ALT is more obvious than that of AST. This trend was also true in the present study, confirming that we have successfully induced acute/chronic liver injury in mice. Furthermore, it is known that ALT and AST levels are positively correlated with the degree of liver injury.^35^ In the present study, we showed that, under the same condition (CCl_4_‐induced liver injury), the livers of HT mice showed a more severe inflammatory response than the livers of WT mice, which further suggests that low pSmad3C expression may reduce the ability of the liver to resist acute/chronic injury.

During CCl_4_‐induced liver injury, there is a rapid increase in IL‐6 and TNF‐α levels, which is regarded as an indication of an inflammatory response.^36^ With the metabolism of various enzymes in the liver, CCl_4_ can stimulate Kupffer cells to secrete factors like IL‐6 and TNF‐α to participate in the inflammatory response.^37^ This inflammatory action is actually a protective measure that promotes hepatocyte regeneration and helps to prevent liver damage. The intensity of the inflammatory response reflects the severity of liver damage. The results presented in the current study show that during liver injury, IL‐6 and TNF‐α levels were increased in both WT and HT mice. However, this was more obvious in HT mice, suggesting that low pSmad3C expression can promote an increase in inflammatory factors during the early stages of liver injury, which then further aggravates liver damage. The relationship between inflammatory factors and the TGF‐β/Smad3 signalling pathway is also associated with activation of Stat3, a cell proliferation and survival regulator.^38^ However, the mechanism of action between pSmad3C and inflammatory factors is complex and requires further exploration.

Our assessment of liver pathology, as well as changes in serum transaminase and inflammatory factors, showed that the degree of liver injury after CCl_4_ stimulation was more severe in HT mice than in WT mice. Western blot data showed that pSmad3C protein expression was significantly increased in the liver tissue of WT mice, but remained low in HT mice. During liver injury, TGF‐β_1_ levels become elevated throughout the body, and participate in various anti‐injury reactions^39^, ^40^ and also up‐regulate pSmad3C protein expression to inhibit hepatocyte proliferation.^41^, ^42^ Therefore, we believe that a mutation in the C‐terminal phosphorylation site of *Smad3*, as in HT mice, affects Smad3 C‐terminal gene coding, resulting in low pSmad3C protein expression. All this then interferes with the anti‐injury effects of the TGF‐β/Smad3 signalling pathway, which could explain why liver damage is more severe in HT mice than in WT mice.

There was no significant difference in phenotype or pSmad3C protein expression between HT and WT mice in the absence of reagent stimulation. However, hepatic lesions were more severe in CCl_4_‐induced liver injury in HT mice than in WT mice, and pSmad3C protein expression was also lower in these HT mice. This suggests that Smad3 C‐terminal phosphorylation may have a protective effect during the early stages of liver injury. Nevertheless, its specific regulatory mechanism is still unclear, and we will explore it in the further. In summary, we have developed a new animal model that will help future research on the effects of domain‐specific phosphorylation of Smad3 during liver disease progression.

## CONFLICT OF INTEREST

There is no conflict of interest.

## Data Availability

The data that support the findings of this study are available from the corresponding author upon reasonable request.

## References

[1] Walton KL , Johnson KE , Harrison CA . Targeting TGF‐beta mediated SMAD signaling for the prevention of fibrosis. Front Pharmacol. 2017;8:461. 28769795 10.3389/fphar.2017.00461 PMC5509761

[2] Chen L , Yang T , Lu DW , et al. Central role of dysregulation of TGF‐beta/Smad in CKD progression and potential targets of its treatment. Biomed Pharmacother. 2018;101:670‐681. 29518614 10.1016/j.biopha.2018.02.090

[3] Boye A , Wu C , Jiang Y , et al. Compound Astragalus and *Salvia miltiorrhiza* extracts modulate MAPK‐regulated TGF‐beta/Smad signaling in hepatocellular carcinoma by multi‐target mechanism. J Ethnopharmacol. 2015;169:219‐228. 25934513 10.1016/j.jep.2015.04.013

[4] Lee YA , Wallace MC , Friedman SL . Pathobiology of liver fibrosis: a translational success story. Gut. 2015;64:830‐841. 25681399 10.1136/gutjnl-2014-306842 PMC4477794

[5] Hu HH , Chen DQ , Wang YN , et al. New insights into TGF‐beta/Smad signaling in tissue fibrosis. Chem Biol Interact. 2018;292:76‐83. 30017632 10.1016/j.cbi.2018.07.008

[6] Matsuzaki K . Smad phosphoisoform signaling specificity: the right place at the right time. Carcinogenesis. 2011;32:1578‐1588. 21798854 10.1093/carcin/bgr172 PMC3204345

[7] Deng Y‐R , Yoshida K , Jin QL , et al. Reversible phospho‐Smad3 signalling between tumour suppression and fibrocarcinogenesis in chronic hepatitis B infection. Clin Exp Immunol. 2014;176:102‐111. 24372395 10.1111/cei.12259 PMC3958159

[8] Yoshida K , Murata M , Yamaguchi T , Matsuzaki K . TGF‐beta/Smad signaling during hepatic fibro‐carcinogenesis (review). Int J Oncol. 2014;45:1363‐1371. 25050845 10.3892/ijo.2014.2552 PMC4151811

[9] Zawel L , Le Dai J , Buckhaults P , et al. Human Smad3 and Smad4 are sequence‐specific transcription activators. Mol Cell. 1998;1:611‐617. 9660945 10.1016/s1097-2765(00)80061-1

[10] He W , Cao T , Smith DA , et al. Smads mediate signaling of the TGFbeta superfamily in normal keratinocytes but are lost during skin chemical carcinogenesis. Oncogene. 2001;20:471‐483. 11313978 10.1038/sj.onc.1204117

[11] Shi Y , Massague J . Mechanisms of TGF‐beta signaling from cell membrane to the nucleus. Cell. 2003;113:685‐700. 12809600 10.1016/s0092-8674(03)00432-x

[12] Feng XH , Derynck R . Specificity and versatility in tgf‐beta signaling through Smads. Annu Rev Cell Dev Biol. 2005;21:659‐693. 16212511 10.1146/annurev.cellbio.21.022404.142018

[13] Pardali K , Kurisaki A , Moren A , et al. Role of Smad proteins and transcription factor Sp1 in p21(Waf1/Cip1) regulation by transforming growth factor‐beta. J Biol Chem. 2000;275:29244‐29256. 10878024 10.1074/jbc.M909467199

[14] Frederick JP , Liberati NT , Waddell DS , et al. Transforming growth factor beta‐mediated transcriptional repression of c‐myc is dependent on direct binding of Smad3 to a novel repressive Smad binding element. Mol Cell Biol. 2004;24:2546‐2559. 14993291 10.1128/MCB.24.6.2546-2559.2004 PMC355825

[15] Chen CR , Kang Y , Massague J . Defective repression of c‐myc in breast cancer cells: a loss at the core of the transforming growth factor beta growth arrest program. Proc Natl Acad Sci USA. 2001;98:992‐999. 11158583 10.1073/pnas.98.3.992 PMC14697

[16] Seoane J , Le H‐V , Shen L , et al. Integration of Smad and Forkhead pathways in the control of neuroepithelial and glioblastoma cell proliferation. Cell. 2004;117:211‐223. 15084259 10.1016/s0092-8674(04)00298-3

[17] Moustakas A , Heldin CH . Non‐Smad TGF‐beta signals. J Cell Sci. 2005;118:3573‐3584. 16105881 10.1242/jcs.02554

[18] Wang JC , Su CC , Xu JB , et al. Novel microdeletion in the transforming growth factor beta type II receptor gene is associated with giant and large cell variants of nonsmall cell lung carcinoma. Genes Chromosomes Cancer. 2007;46:192‐201. 17117417 10.1002/gcc.20400

[19] Murata M , Matsuzaki K , Yoshida K , et al. Hepatitis B virus X protein shifts human hepatic transforming growth factor (TGF)‐beta signaling from tumor suppression to oncogenesis in early chronic hepatitis B. Hepatology. 2009;49:1203‐1217. 19263472 10.1002/hep.22765

[20] Hu X , Rui W , Wu C , et al. Compound Astragalus and *Salvia miltiorrhiza* extracts suppress hepatocarcinogenesis by modulating transforming growth factor‐beta/Smad signaling. J Gastroenterol Hepatol. 2014;29:1284‐1291. 24329763 10.1111/jgh.12490

[21] Millet C , Zhang YE . Roles of Smad3 in TGF‐beta signaling during carcinogenesis. Crit Rev Eukaryot Gene Expr. 2007;17:281‐293. 17725494 10.1615/critreveukargeneexpr.v17.i4.30 PMC2639747

[22] Matsuzaki K , Seki T , Okazaki K . TGF‐beta signal shifting between tumor suppression and fibro‐carcinogenesis in human chronic liver diseases. J Gastroenterol. 2014;49:971‐981. 24263677 10.1007/s00535-013-0910-2

[23] Yoshida K , Murata M , Yamaguchi T , et al. Reversible human TGF‐ signal shifting between tumor suppression and fibro‐carcinogenesis: implications of Smad phospho‐isoforms for hepatic epithelial‐mesenchymal transitions. J Clin Med. 2016;5:7. 10.3390/jcm5010007 PMC4730132 26771649

[24] Sigmund CD . Major approaches for generating and analyzing transgenic mice. An overview. Hypertension. 1993;22:599‐607. 8406666 10.1161/01.hyp.22.4.599

[25] Fujii W . Generation of knock‐in mouse by genome editing. Methods Mol Biol. 2017;1630:91‐100. 28643252 10.1007/978-1-4939-7128-2_8

[26] Yang X , Letterio JJ , Lechleider RJ , et al. Targeted disruption of SMAD3 results in impaired mucosal immunity and diminished T cell responsiveness to TGF‐beta. EMBO J. 1999;18:1280‐1291. 10064594 10.1093/emboj/18.5.1280 PMC1171218

[27] Zhu Y , Richardson JA , Parada LF , Graff JM . Smad3 mutant mice develop metastatic colorectal cancer. Cell. 1998;94:703‐714. 9753318 10.1016/s0092-8674(00)81730-4

[28] Weinstein M , Yang X , Deng C . Functions of mammalian Smad genes as revealed by targeted gene disruption in mice. Cytokine Growth Factor Rev. 2000;11:49‐58. 10708952 10.1016/s1359-6101(99)00028-3

[29] Wu JJ , Jiang YF , Wu C , et al. Construction and functional study of three plasmids including Smad3 WT, Smad3 EPSM and Smad3 3S‐A stably transfected HepG2 cell lines. Chin Pharmacol Bull. 2016;32:825‐831.

[30] Sekimoto GO , Matsuzaki K , Yoshida K , et al. Reversible Smad‐dependent signaling between tumor suppression and oncogenesis. Cancer Res. 2007;67:5090‐5096. 17545585 10.1158/0008-5472.CAN-06-4629

[31] Heyer J , Escalante‐Alcalde D , Lia M , et al. Postgastrulation Smad2‐deficient embryos show defects in embryo turning and anterior morphogenesis. Proc Natl Acad Sci USA. 1999;96:12595‐12600. 10535967 10.1073/pnas.96.22.12595 PMC23005

[32] Ullah I , Sun W , Tang L , Feng J . Roles of Smads family and alternative splicing variants of Smad4 in different cancers. J Cancer. 2018;9:4018‐4028. 30410607 10.7150/jca.20906 PMC6218760

[33] Yang X , Li C , Xu X , Deng C . The tumor suppressor SMAD4/DPC4 is essential for epiblast proliferation and mesoderm induction in mice. Proc Natl Acad Sci USA. 1998;95:3667‐3672. 9520423 10.1073/pnas.95.7.3667 PMC19893

[34] Lee HS , Kim HH , Ku SK . Hepatoprotective effects of *Artemisiae capillaris* herb and Picrorhiza rhizome combinations on carbon tetrachloride‐induced subacute liver damage in rats. Nutr Res. 2008;28:270‐277. 19083419 10.1016/j.nutres.2008.02.001

[35] Tan KK , Bang SL , Vijayan A , Chiu MT . Hepatic enzymes have a role in the diagnosis of hepatic injury after blunt abdominal trauma. Injury. 2009;40:978‐983. 19535055 10.1016/j.injury.2009.02.023

[36] Zhang D‐G , Zhang C , Wang J‐X , et al. Obeticholic acid protects against carbon tetrachloride‐induced acute liver injury and inflammation. Toxicol Appl Pharmacol. 2017;314:39‐47. 27865854 10.1016/j.taap.2016.11.006

[37] Hines IN , Kremer M , Isayama F , et al. Impaired liver regeneration and increased oval cell numbers following T cell‐mediated hepatitis. Hepatology. 2007;46:229‐241. 17596893 10.1002/hep.21674

[38] Moh A , Iwamoto Y , Chai G‐X , et al. Role of STAT3 in liver regeneration: survival, DNA synthesis, inflammatory reaction and liver mass recovery. Lab Invest. 2007;87:1018‐1028. 17660847 10.1038/labinvest.3700630

[39] Niu L , Cui X , Qi Y , et al. Involvement of TGF‐beta1/Smad3 signaling in carbon tetrachloride‐induced acute liver injury in mice. PLoS ONE. 2016;11:e0156090. 27224286 10.1371/journal.pone.0156090 PMC4880333

[40] Yoshida K , Matsuzaki K . Differential regulation of TGF‐beta/Smad signaling in hepatic stellate cells between acute and chronic liver injuries. Front Physiol. 2012;3:53. 22457652 10.3389/fphys.2012.00053 PMC3307138

[41] Dooley S , ten Dijke P . TGF‐beta in progression of liver disease. Cell Tissue Res. 2012;347:245‐256. 22006249 10.1007/s00441-011-1246-y PMC3250614

[42] Akhmetshina A , Palumbo K , Dees C , et al. Activation of canonical Wnt signalling is required for TGF‐beta‐mediated fibrosis. Nat Commun. 2012;3:735. 22415826 10.1038/ncomms1734 PMC3316881

